# Coactivator Recruitment of AhR/ARNT1

**DOI:** 10.3390/ijms150611100

**Published:** 2014-06-19

**Authors:** Alexander Endler, Li Chen, Futoshi Shibasaki

**Affiliations:** Department of Molecular Medical Research, Tokyo Metropolitan Institute of Medical Science, 2-1-6 Kamikitazawa, Setagaya-ku, Tokyo 156-8506, Japan; E-Mail: LiCHEN@biomed-sh.com

**Keywords:** SRC1, NCoA2, AhR, ARNT, TCDD, ER, AR

## Abstract

A common feature of nuclear receptors (NRs) is the transformation of external cell signals into specific transcriptions of the signal molecule. Signal molecules function as ligands for NRs and, after their uptake, activated NRs form homo- or heterodimers at promoter recognition sequences of the specific genes in the nucleus. Another common feature of NRs is their dependence on coactivators, which bridge the basic transcriptional machinery and other cofactors to the target genes, in order to initiate transcription and to unwind histone-bound DNA for exposing additional promoter recognition sites via their histone acetyltransferase (HAT) function. In this review, we focus on our recent findings related to the recruitment of steroid receptor coactivator 1 (SRC1/NCoA1) by the estrogen receptor-α (ERα) and by the arylhydrocarbon receptor/arylhydrocarbon receptor nuclear translocator 1 (AhR/ARNT1) complex. We also describe the extension of our previously published findings regarding the binding between ARNT1.1 exon16 and SRC1e exon 21, via *in silico* analyses of androgen receptor (AR) NH2-carboxyl-terminal interactions, the results of which were verified by *in vitro* experiments. Based on these data, we suggest a newly derived tentative binding site of nuclear coactivator 2/glucocorticoid receptor interacting protein-1/transcriptional intermediary factor 2 (NCOA-2/ GRIP-1/TIF-2) for ARNT1.1 exon 16. Furthermore, results obtained by immunoprecipitation have revealed a second leucine-rich binding site for hARNT1.1 exon 16 in SRC1e exon 21 (LSSTDLL). Finally, we discuss the role of 2,3,7,8-tetrachlorodibenzo-*p*-dioxin (TCDD) as an endocrine disruptor for estrogen related transcription.

## 1. Basic Signal Transmission Mechanisms of Nuclear Receptors

Nuclear receptors can be categorized not only based on the nature of their DNA binding sites, but also according to whether they form homodimers, such as the estrogen receptor (ER), progesterone receptor (PR), androgen receptor (AR), glucocorticoid receptor (GR), and mineralocorticoid receptor (MR), or heterodimers, such as the retinoic acid receptor (RAR), thyroid hormone receptor (TR), and vitamin D3 receptor (VDR). The latter one interact with the receptor for 9-cis retinoic acid (RXR) [1]. A common characteristic of all NRs is that, after penetration of hydrophobic chemicals through the cell membrane, they incorporate their specific ligands into the ligand binding domains (LBD), which are highly variable within the NRs, due to their ligand specificities [2]. In the absence of ligands, cytosolic NRs are coupled to heat shock protein hsp90, hsp70, Hop, hsp40, and p23 multi-protein complexes [3], which enables them to bind ligands. In contrast RAR, TR, and VDR reside in the nucleus, with binding only to corepressors in their inactive state.

After agonist uptake, NRs undergo dimer formation, leading to accessibility of coactivators and release of corepressor proteins. The canonical pathway for coactivator recruitment of NRs, after agonist binding in their LBDs, is a helix 12 realignment with helices 3, 5/6, and 11, which, thereby, form a lid on the LBD for the engulfed ligands. The LBD contains a conserved activation function 2 (AF2) domain, which is in the ligand activated conformation the connective link to LXXLL domains in the central nuclear receptor interaction domain (NID) of steroid receptor coactivators (SRCs), also called the p160 SRC family [4]. SRCs interact with NRs as coactivators and facilitate their transcription via their histone acetylase (HAT) activity [5,6]. In addition, the activation domain 1 (AD1) of SRCs binds to the c-terminal SRC interaction domain (SID) of p300, as well as its homolog, the cAMP response element-binding (CREB) protein (CBP) [7]. The p300/CBP complex plays an important role in the transcription process through connecting other transcriptional activators, basal transcription factors, and HATs to the transcriptional machinery [8].

## 2. AhR/ARNT-Related Transcription

The aryl hydrocarbon receptor (AhR), which is, like ARNT, a member of the basic helix-loop-helix (bHLH) Per-ARNT-SIM (PAS) family, recognizes the halogenated aromatic hydrocarbon 2, 3, 7, 8-Tetrachlorodibenzodioxin (TCDD) or the polycyclic aromatic hydrocarbon (PAH) 3-methylcholanthrene (3MC) as ligands. Unliganded AhR resides in the cytosol and forms a complex with hsp90, hsp23 [9], AhR-activated 9 (ARA9) [10], and hepatitis B virus X-associated protein 2 (XAP2), which is similar to immunophilin [11]. After ligand uptake, AhR forms an AhR/ARNT1 complex with its obligate partner ARNT1 and transcribes cytochrome P450s (Phase I), as well as UDP-glucuronosyltransferase and glutathione-*S*-transferase (GST) (Phase II) via xenobiotic responsive elements (XREs) within their promoter regions [12]. Unlike other NRs, a unique major endogenous ligand for the AhR has not been established, but the following ligands have been reported as agonists: tryptophan (Trp) catabolite kynurenine (Kyn) [13], indigo and indirubin [14], bilirubin [15], and prostaglandins [16]. According to a hypothesis developed in the early 1990s, enzymes of the cytochrome P450 family originally evolved as animal-plant “warfare” enzymes to protect themselves from toxic chemicals produced by plants [17]. Later, Denison and Whitlock proposed that pathways of the cytochrome P450 enzymes help to maintain homeostasis of endogenous lipophilic substances [18,19].

## 3. AhR-Coactivator Interactions

The AhR is a ligand-induced transcription factor but lacks an AF2 domain. Since the AF2 domain is the common link between transcription factors and the SRCs, the question arose how the AhR is recruiting coactivators. Kim *et al*. described a coiled-coil coactivator (CoCoA), which is a secondary coactivator for NRs interacting with the bHLH-PAS domain of p160 proteins, but also functions as a potent primary coactivator for AhR/ARNT transcription also via bHLH-PAS domain interaction, underlining, that AhR/ARNT can recruit several coactivators [20]. The authors noted that AhR bound CoCoA in a ligand-independent manner, and suggested that CoCoA may exist in a cytosolic complex with AhR before ligand activation, and travels with AhR to its target promoter after ligand binding. Others demonstrated a TCDD and LXXLL motif-dependent SRC1 interaction with a Q-rich subdomain of the AhR [21]. Notably, the transcriptional activity of the AhR also depends on the type of ligand. In the case of AhR agonist application, ordinary transcription with cyclic recruitment of NCOA1, NCOA2, and NCOA3 (each present on the promoter) simultaneously occurs with concomitant enhanced *CYP1A1* transcription. In contrast, the AhR antagonist 3,3'-diindolylmethane (DIM) promotes AhR nuclear translocation and p160 coactivator recruitment, but fails to recruit Pol II or cause histone acetylation, suggesting that ligand-dependent changes in AhR conformation can affect its transcriptional activity [22].

## 4. Coactivator Recruitment to the AhR/ARNT Complex

**Figure 1:**
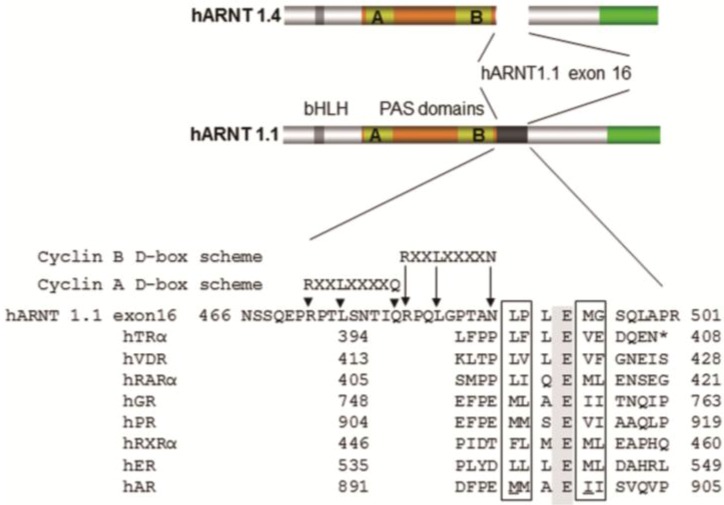
Scheme of AF2 domains. The boxes indicate hydrophobic amino acids beside the conserved glutamic acid. Underlined amino acids in hAR indicate hydrophobic residues, which serve as coactivator interface [25].

**Figure 2:**
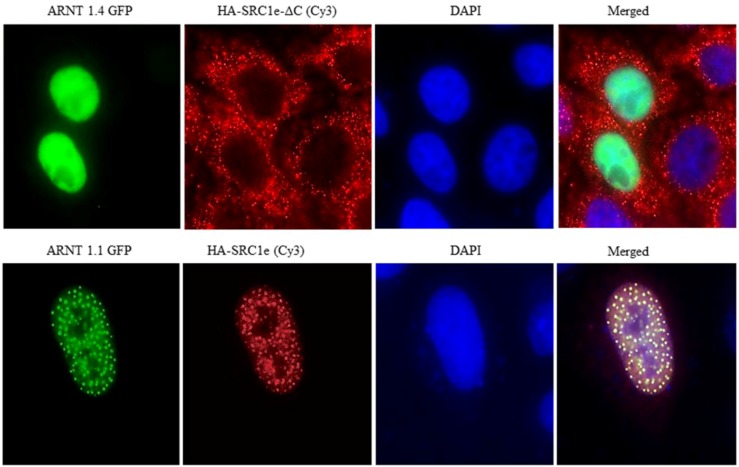
HeLa cells after cotransfection with the indicated hARNT1.1, hARNT1.4, SRC1e, and SRC1e-ΔC constructs. Green indicates GFP, red indicates HA-CY3 staining, and blue indicates nuclear DAPI staining (adapted from [23]). Twenty-four hours after transfection, fixed cells were incubated with HA antibodies (12CA5 hybridomas, MBL) for 1 h, washed, and then incubated with Cyanine 3 (Cy3)-conjugated secondary antibodies for 30 min. The cells were then washed again and mounted in Vectashield (Vector Laboratories, Burlingame, CA, USA) mounting medium containing 4,6-diamidino-2-phenylindole (DAPI). Fluorescence images were visualized using an Olympus 1670 inverted system microscope (Olympus Optical Co., Ltd, Tokyo, Japan) equipped with a charge-coupled device (Magnification ×1000).

In a recent publication, we described an AF2 domain in ARNT1 (Figure 1) and suggested that the AhR/ARNT1 complex is a transcription factor complex in which the LBD and the AF2 domain are distributed on two factors [23]. For complete transcriptional activity of the AhR/ARNT1 complex, two cyclin degradation boxes adjacent to the ARNT1 AF2 domain are necessary, which lead to accelerated *CYP1A1* transcription (for transcription factor activation via degradation, refer to the review by Lipford and Deshaies [24]). We found, that the AF2 domains of both ARNT1 and ERα- bind to the exon 21 of SRC1e, and these interactions are crucial for TCDD- and 17β-estradiol (E2)-related transcriptions. ARNT1 exists as a splice variant (ARNT1.4) without exon 16, which contains the AF2 domain, and SRC1e also exists as a splice variant without the binding site for ARNT1-AF2 and ER-AF2 domains on exon 21 (SRC1e-ΔC). Cotransfection of SRC1e with ARNT1.1 showed co-localization in the nuclei of HeLa cells (Figure 2), whereas both ARNT1 splice variants located in the nucleus and both SRC1s in the cytosol without the interaction. SRC1e overexpression alone did not lead to maximum transcription enhancement, which needed cotransfection with ARNT1.1 for TCDD and either ARNT1.1 or ARNT1.4 for E2 responses. This observation revealed that SRC1e and ARNT.1 depend on each other for maximum transcriptional activities in MCF7 cells. Furthermore, a comparison of ARNT1.1 and ARNT1.4 transcription patterns showed, that both splice variants led to similar activities regarding hypoxia and ER-related responses, whereas TCDD transcription was dependent on the intact exon 16 of ARNT1.1 [23].

**Figure 3:**
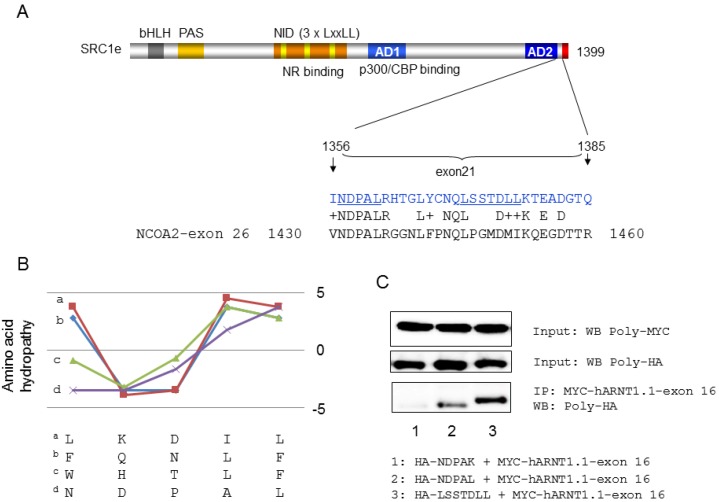
Evaluation of the hARNT1.1 AF-2 binding site in SRC1e exon 21. (**A**) Scheme of SRC1e and amino acid composition of exon 21 (upper image) and alignment with NCOA2 exon 26. Underlined aa are the binding sites for hARNT1.1 AF-2; (**B**) Comparison of hydropathy pattern between *C*-terminal binding peptides for AF2 bindings in hAR [26] and the common NDPAL sequence in exon 21 of SRC1e and exon 26 of NCOA2; (**C**) Analysis of hARNT1.1 and SRC1e exon 16 interactions with immunoprecipitation of HA- tagged GFP-SRC1e-exon 21 aa stretches and MYC-tagged GFP-hARNT1.1-exon 16 with MYC-beads. **Upper** panel: WB of MYC-tagged GFP-hARNT1.1-exon 16 after MYC-beads immunoprecipitation with poly-MYC antibodies; **Middle** panel: WB of indicated HA-tagged GFP-SRC1e-exon 21 aa stretches with poly-HA antibodies (cell lysates); **Lower** panel: WB after MYC-beads immunoprecipitation with poly HA antibodies. Abbreviations: PAS, Per/ARNT/SIM domain; bHLH, basic helix-loop-helix domain; NID, nuclear receptor interaction domain; AD1 and AD2, activation domain 1 and activation domain 2. (Transfected HeLa cells were lysed and the supernatant was incubated with anti-myc-conjugated agarose (Sigma-Aldrich, St. Louis, MO, USA) overnight at 4 °C. The resulting precipitate was washed with RIPA buffer and then boiled in 2xSDS sample buffer for 10 min. Co-immunoprecipitated HA tagged NDPAL, NDPAK, and LSSTDLL were analyzed by Western blotting with anti-HA-polyclonal antibodies (Sigma-Aldrich, St. Louis, MO, USA) followed by horseradish-peroxidase-conjugated anti-rabbit IgG (Thermo Fisher Scientific Inc., Rockford, IL, USA) incubation for 1 h. Immunoprecipitate analysis was done using a SuperSignal West Dura chemiluminescent detection system (Thermo Fisher Scientific Inc., Rockford, IL, USA).

For this review, we further analyzed the binding of SRC1e-exon 21 and the AF2 domain of ARNT1.1. As shown in Figure 3A, the alignment of SRC1e-exon 21 and NCOA2-exon 26 showed a common peptide with the amino acid sequence NDPALR. Because the binding between ARNT1 and SRC1e is LXXLL motif independent, we investigated other LXXLL independent interfaces for NR-AF2 domains. After ligand binding, the AR exhibits an amino-carboxy terminal interaction, and a similar mechanism has been postulated for the PR [27]. For the AR, the three amphipathic ^23^FQNLF^27^, ^179^LKDIL^183^, and ^432^WHTLF^436^ a-helices within the AR interact with its AF2 hydrophobic groove. Particularly, FQNLF has a five-fold higher affinity for the AR-AF2 domain than for LXXLL motifs in the NID of SRCs in response to ligand binding. The AR N-terminus also interacts with cyclin D1, which inhibits the FQNLF-AF2 interaction and leads to reduced transcriptional activity [26]. After ligand-induced folding, the AR amino-terminal domain (NTD) interacts with coactivators in a NID-independent fashion, as shown by LXXLL mutations in SRC1e and NCOA2 [28]. Regarding the hydropathy pattern of FQNLF, LKDIL, and WHTLF, the second and third amino acids are hydrophilic, whereas the fourth and fifth are hydrophobic, which also occurs in NDPAL (Figure 3B). Further analysis via immunoprecipitation (IP) revealed a binding between NDPAL and exon 16 of ARNT 1, whereas changing NDPAL to NDPAK, in which K is a highly hydrophilic amino acid, abrogated the interaction (Figure 3C). During the immunoprecipitation (IP) measurements, control IPs of the remaining SRC1 exon 21 revealed a second binding interface for hARNT1.1-exon 16, which we could identify as LSSTDLL (Figure 3C). Since NDPAL is common in SRC1 and NCOA2, we propose that the same mechanism of AF2–NDPAL binding might also occur with NCOA2, because NCOA2 is also a coactivator of AHR/ARNT1-related [29] and E2-related [30] transcriptions. However, the other binding site (LSSTDLL) for the hARNT1.1 AF2 domain is specific for SRC1. None of the binding peptides are present on SRC3. In humans, 48 nuclear hormone receptors are described [31], and since the AF-2 domains of ARNT1.1 and ERα both interact with SRC1e exon 21, the question is whether other NRs also use the binding interface on SRC1e (and probably on NCOA2) for interactions, and what role the SRC1e-ΔC plays.

## 5. The Role of TCDD as Endocrine Disrupter

In contrast to 3MC, which is fast degraded into carcinogenic intermediates by the activity of the AhR/ARNT complex [32], TCDD is a stable chemical, with a half-life of 15.4 months in humans [33]. Therefore, dioxin poses an extremely long-lasting challenge for the cells. Targeted degradation via the ubiquitin–proteasome pathway is thought to be a cause of TCDD-induced endocrine disruption, because TCDD initially induces the formation of a nuclear AhR complex, which coordinately recruits ERα and the proteasome complex, resulting in the degradation of both receptors [34]. Recruitment of liganded and unliganded ER to XREs by activated AhR has been proposed to modulate AhR signaling, but there are contrary opinions as to whether the interaction is AhR-activating [35] or downregulating [36]. Furthermore, the reverse direction also has been described, in which the unliganded ER recruits 3MC-activated AhR to EREs, leading to estrogen-related transcription [37]. However, 3MC has subsequently been recognized as a mixed AhR/ER agonist [38].

In addition to transrepression by transcription factor recruitment to factor-unrelated promoter areas, coactivator recruitment has been proposed to be a transcription rate-limiting step for the activity of transcription factors [39,40,41]. Together with the hypoxia-inducible factor-1α (HIF-1α), ARNT1, also named hypoxia-inducible factor-1β (HIF-1β), forms the HIF-1 complex and recognizes target genes via hypoxia-responsive elements (HREs) in their promoter regions after oxygen deprivation [42]. In addition, ARNT1 has been shown to be a potent coactivator of ERs, because their LBDs interact after E2 activation with the c-terminal transactivation domain (TAD) of ARNT1 [43], but this seems to be cell line specific, since Labrecque and colleagues showed that, in contrast to MCF7 cells, ARNT exhibited a dioxin-independent co-repressor function for estrogen signaling in human ECC-1 endometrial carcinoma cells [44]. Competition for ARNT was reported to be a transcription rate-limiting step for ER under hypoxia and during TCDD induction [45], because ARNT was sequestered to other promoter areas than the EREs. TCDD-related responses, however, were essentially reduced under hypoxia, but the cause was not ARNT availability limitation [46]. We suggest that ARNT1.4 might represent a minor ARNT1 pool, which maintains complete response to hypoxia but is reduced transcription activating upon dioxin challenge.

The subcellular localization of SRC1 has been described as nuclear immediately after translation and then cytosolic within 48 h [47]. In our previous experiments, we derived a similar pattern with HeLa cells without ARNT1.1 SRC1e binding. With ARNT1.1-SRC1e interaction, however, both proteins aggregated in the nucleus (Figure 2). Small fractions of the ER were shown to be subject of constant nucleocytoplasmic shuttling with agonist and antagonist, as well as SRC1 binding reducing the motility. For SRC1 and unliganded cytosolic ER interactions, the complex is assumed to be degraded in the cytosol [48]. With the assumption that cytosolic SRC1e is degraded [47,48], SRC1e appears to be rescued by ARNT1.1 but not by ARNT1.4 and SRC1e-ΔC, which had no effect on E2-induced transcription, did not accumulate in the nucleus when co-transfected with both ARNT1s [23]. Since both ARNTs and SRC1es exist as splice variants, we propose that there is a selective and ligand-independent ARNT1.1-SRC1e accumulation in the nucleus before assembly of complete transcriptional machineries, which primarily facilitates detoxification but also promotes E2-related transcription. Depending on the AhR ligands, ERs are directed to XREs via AhR interaction, and estrogen response is suppressed by ER occupation and degradation [34]. Since we showed that ERα binds E2 and LXXLL independent to SRC1e, even unliganded ERα might shuttle SRC1e to the activated AhR complex, which supports the hypothesis that AhR-ER interactions enhance CYP1A1 transcription [49]. Depending on the cytochrome susceptibility of the AhR ligands, however, estrogen response might be severely compromised.

## 6. Conclusions

Coactivator recruitment of NRs includes non-canonical protein interactions, which are independent of the LXXLL motifs in the NCOAs, as shown for the AR. The ARNT1.1-AF2 domain on exon 16 binds ligand and LXXLL motif independent to two peptide stretches on exon 21 of SRC1e. One of them is also present on NCOA2 and derived from a comparison with peptides involved in the AR-specific amino-carboxy terminal interactions, whereas the other one is SRC1 specific. The binding leads to nuclear localization of ARNT1.1 and SRC1e, which is essential for TCDD-induced transcription. The ERα AF2 domain also binds to SRC1e exon 21, which is essential for E2-related transcriptional responses. We suggest that there is a selective nuclear SRC1e-ARNT1.1 assembly before ligand inductions, which primarily facilitates detoxification but also promotes E2-related transcription. Since SRC1e is a major co-activating factor for estrogen response, its constant involvement in TCDD detoxification, in combination with AhR-directed degradation of transcriptionally inactive ER, leads to E2-related transcription breakdown.
